# A novel heterozygous mutation in PTHLH causing autosomal dominant brachydactyly type E complicated with short stature

**DOI:** 10.1002/mgg3.2393

**Published:** 2024-02-05

**Authors:** Jian Sun, Nian Yang, Zhengquan Xu, Hongbo Cheng, Xiangxin Zhang

**Affiliations:** ^1^ Center for Reproduction and Genetics, NHC Key Laboratory of Male Reproduction and Genetics, Suzhou Municipal Hospital The Affiliated Suzhou Hospital of Nanjing Medical University Suzhou China; ^2^ Department of Pediatrics LinShu People's Hospital Linyi China; ^3^ Department of Orthopaedics, Suzhou Municipal Hospital The Affiliated Suzhou Hospital of Nanjing Medical University Suzhou China

**Keywords:** brachydactyly type E, mutation, next‐generation sequencing, *PTHLH*

## Abstract

**Background:**

Brachydactyly type E (BDE) is a general term characterized by variable shortening of metacarpals and metatarsals, with phalanges affected frequently. It can occur as an isolated form or part of syndromes and manifest a high degree of phenotypic variability. In this study, we have identified the clinical characteristics and pathogenic causes of a four‐generation pedigree with 10 members affected by BDE and short stature.

**Methods:**

After the informed consent was signed, clinical data and peripheral blood samples were collected from available family members. Karyotype analysis, array‐CGH, next‐generation sequencing, and Sanger sequencing were employed to identity the pathogenic candidate gene.

**Results:**

No translocation or microdeletion/duplication was found in karyotype analysis and array‐CGH; hence, a novel heterozygous mutation, c.146dupA. p.S50Vfs*22, was detected by next‐generation sequencing in *PTHLH* gene, leading to a premature stop codon. Subsequently, the mutation was confirmed by Sanger sequencing and co‐segregation analysis.

**Conclusion:**

In this study, we described a novel heterozygous mutation (c.146dupA. p.S50Vfs*22) of gene *PTHLH* in a Chinese family. The mutation could induce a premature stop codon leading to a truncation of the protein. Our study broadened the mutation spectrum of *PTHLH* in BDE.

## Graphical abstract

(A) No obvious chromosome aberration was found in the affected individuals. (B) Direct sequencing of PTHLH exons showing heterozygous c.146dupA, p.S50Vfs*22 mutation identified in BDE affected individuals (II2, III6, III16, III17, IV11) but not in normal family members (III5, IV2). (C) Evolutionary conservation of the cluster across multiple species.

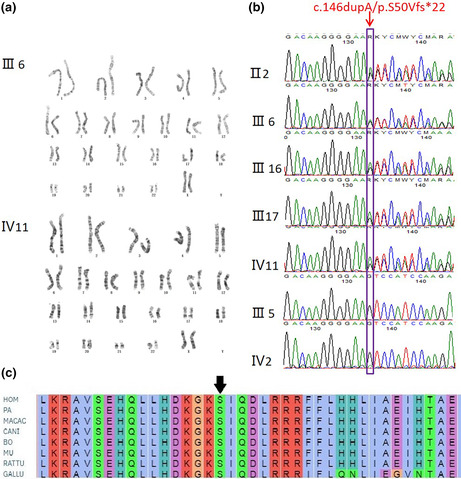

## 1 INTRODUCTION

Brachydactyly (BD) is a general term characterized by disproportionately short fingers and toes, and five types (A–E) have been classified based on anatomic classification (Temtamy & Aglan, 2008). Of which, brachydactyly type E (BDE, OMIM #113300) is a rare syndrome and encompasses variable shortening of metacarpals and metatarsals, with phalanges affected frequently (Mundlos, 2009). Hertzog has classified BDE into three types, which include type E1, with shortening of metacarpal IV, sometimes involving the shortening of metatarsal IV; type E2, with shortening of metacarpals IV and V, and/or metatarsals, associated with shortening of the distal phalanx of the thumb and type E3, associated with variable combinations of short metacarpals, without phalangeal (Hertzog, 1968). However, these patterns are not exhibited exactly in all syndromes, and constantly manifest a high intrafamilial and interindividual phenotypic variability.

The majority of BDE cases occur as a part of multiple syndromes, such as hypertension‐brachydactyly syndrome, Turner syndrome, Bilginturan BD, brachydactyly mental retardation syndrome, and BDE with short stature (Pereda et al., 2013).

And most BDE isolated affected individuals are inherited in an autosomal dominant pattern. To date, the etiology of BDE has been investigated intensively and chromosome translocation, microdeletion/duplication syndromes, and point mutation of HOXD13 and *PTHLH* genes have been reported to be related to the syndrome (Flottmann et al., 2016; Johnson et al., 2003; Klopocki et al., 2010; Maass et al., 2010). However, the genetic cause of the great majority of BDE cases remains unexplained due to the lack of sufficient cases.

In this study, we described a four‐generation Chinese family with a novel pathogenic variant c.146dupA. p.S50Vfs*22 in gene *PTHLH*, presenting with BDE complicated with short stature. The variant could lead to a truncation of the protein and subject to nonsense‐mediated decay which enriched the spectrum of *PTHLH* gene mutations in BDE.

## 2 MATERIALS AND METHODS

### 2.1 Ethical compliance

This study was approved by the institutional ethics committee of the Affiliated Suzhou Hospital of Nanjing Medical University, the reference number is K‐2022‐208‐K01, and written informed consent was obtained from every participant in the study.

### 2.2 Karyotype analysis and array‐CGH

G‐banded karyotyping was performed on peripheral blood leukocytes according to routine procedures; high‐resolution oligonucleotide array‐CGH was performed on Agilent Human CGH 44 K Oligo Microarrays, in accordance with standard and manufacturer's recommendations.

### 2.3 Whole‐exome sequencing

DNA from peripheral blood was extracted using standard methods. Exome captured sequencing library was produced from xGen Exome Research Panel v1.0 kit. The libraries were subsequently sequenced on an Illumina HiSeq X‐ten sequencing instrument according to the manufacturer's instructions. After sequencing, the short reads were compared with Genome Reference Consortium Human genome build 37(GRCh37). Sequence Alignment/Map (SAM) tools software (v0.1.18) was used to align these short sequences. The Genome Analysis Toolkit (GATK) software (v2.6–4) was used to seek sample sequencing data and the difference loci (SNP and InDel) of the referred genome, while Annovar software (2013Aug23 version) was applied for functional annotation of these mutation sites. Additionally, the Exome Aggregation Consortium (ExAC Version 0.3.1), Genomes 1000 Project, ESP6500, and other public database were performed to filter the mutations. Compared with the East Asian population of ExAC, we retained the mutations in autosomal dominant or X‐linked dominant inheritance whose frequency was less than 0.01, and retained mutations in autosomal recessive or X‐linked recessive inheritance whose frequency was less than 0.05 in correspondence. After the removal of the synonymous mutation loci that have no effect on splicing, and with the application of Sorting Intolerant from Tolerant (SIFT), polyphen2, and other software, we predicted pathogenicities of the remaining loci in exon and the domain of the 20 bp around exon. The causative mutation sites were selected according to the disease phenotype and pedigree information.

### 2.4 Sanger sequencing validation

Sanger sequencing and co‐segregation analysis of other family member samples were used to confirm the candidate variants. The sequence containing the mutation was amplified by PCR with the primers.

## 3 RESULTS

### 3.1 Clinical features

**FIGURE 1:**
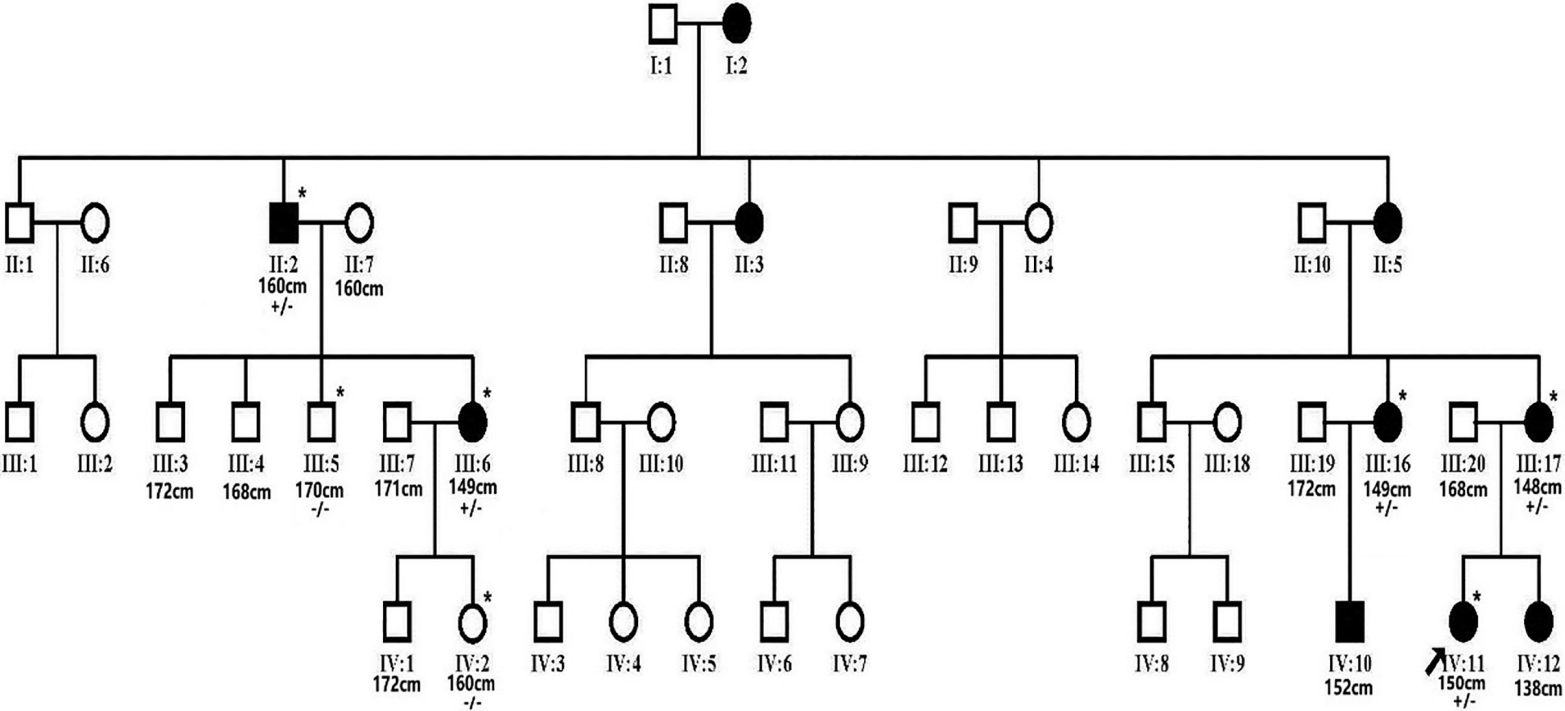
Pedigree of the Chinese family. Brachydatylies are found in every generation in both male and female indicating autosomal dominant trait of inheritance. Proband is denoted with an arrow. The asterisks in the pedigree indicate *PTHLH* resequencing of family members. +/− represents the heterozygous *PTHLH*, p.S50Vfs*22 variant, −/− represents the wild type.

A four‐generation pedigree with autosomal dominant brachydactyly from Shandong Province, China, was enrolled in the study (Figure 1). There are 44 surviving members in the family, including 10 patients, 34 normal individuals.

**FIGURE 2:**
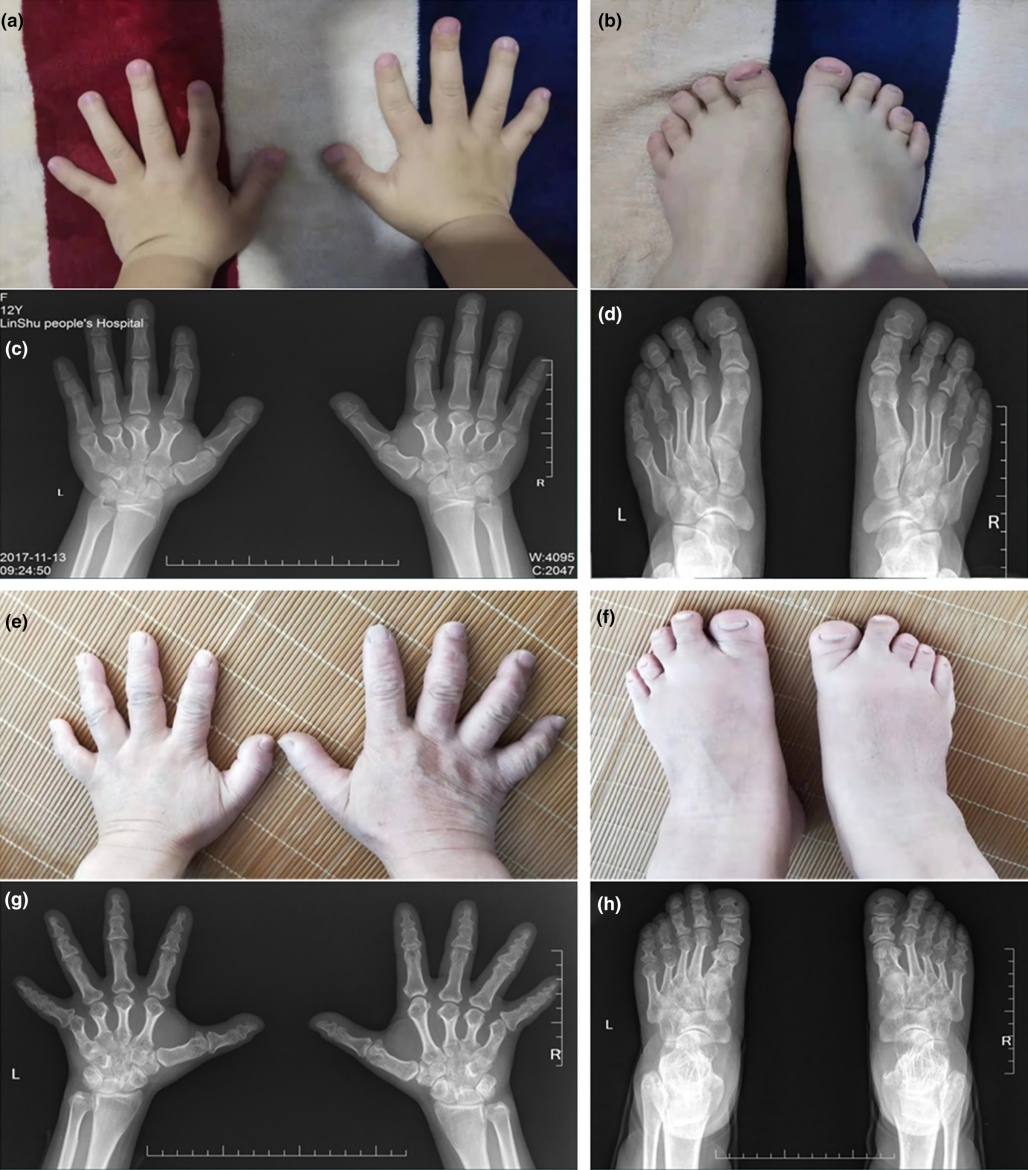
Clinical and radiological features observed in the proband (IV11) in the age of 17 (a–d) as well as III6 at the age of 32 years (e–h). (a) Clinically observed bulky and stocky hands. (b) Shortening of fourth and fifth toes. (c) Hand radiograph showing shortened metacarpals and phalanges as well as abnormal cone‐shaped epiphyses of the metacarpals. (d) Shortening fourth and fifth metatarsals of both feet. (e) Clinically observed short and stubby hands. (f) Shortening of third, fourth, and fifth toes in right foot, shortening of third, fourth in left foot. (g) Radiologically observed shortened metacarpals and phalanges as well as abnormal cone‐shaped epiphyses of the metacarpals. (h) Foot radiograph showing shortened third, fourth, and fifth metatarsals in right foot and shortened third, fourth metatarsals in left foot.

The proband (IV11) was a 17‐year‐old female, her physical examination presented with short and broad hands, shortened fourth and fifth toes in both feet. Moreover, her stature was short with a height of 150 cm (<1 SD). The radiographic images of her hands revealed that all the metacarpals and phalanges of both hands were shortened with premature fusion of the epiphyses in the shortened bones and cone‐shaped epiphyses. The feet radiographic images exhibited shortening fourth and fifth metatarsals of both feet (Figure 2a–d). Overall, the phenotype resembled BDE (Brachydactyly type E, OMIM #113300) with additional features of short stature. Patient III6, who was 149 cm tall (−2 SD), was a 32‐year‐old female who presented with similar symptoms in hands comparing with the proband, but her feet roentgenogram exhibited shortening of third, fourth, and fifth metatarsals in right foot, and shortening of third, fourth metatarsals in left foot accompanied by expanded epiphysis (Figure 2e–h). Clinical features of other patients were also examined. Patient II2, whose fourth metacarpals were short in both hands, was male and 160 cm tall (−2 SD). Patient III17, whose height was 148 cm (−2 SD), had bilateral shortened metacarpals and phalanges, especially median phalange of the third finger, and the epiphysis of all metacarpals and some phalanges were expanded. Patient III 16 who was 149 cm (−2 SD) tall, had bilateral shortened metacarpals and phalanges, and the metacarpophalangeal articular surface was irregular in either hand.

However, features of facial abnormalities, learning disabilities, mammary gland or thyroid gland maldevelopment, dental malposition, and oligodontia were not found in this family. Laboratory testing results of the bone metabolism and hormone levels such as PTH, TSH, vitamin D3, alkaline phosphatase, free triiodothyronine (FT3), and free thyroxine (FT4) were normal.

### 3.2 Genetic analysis and expression studies

**FIGURE 3:**
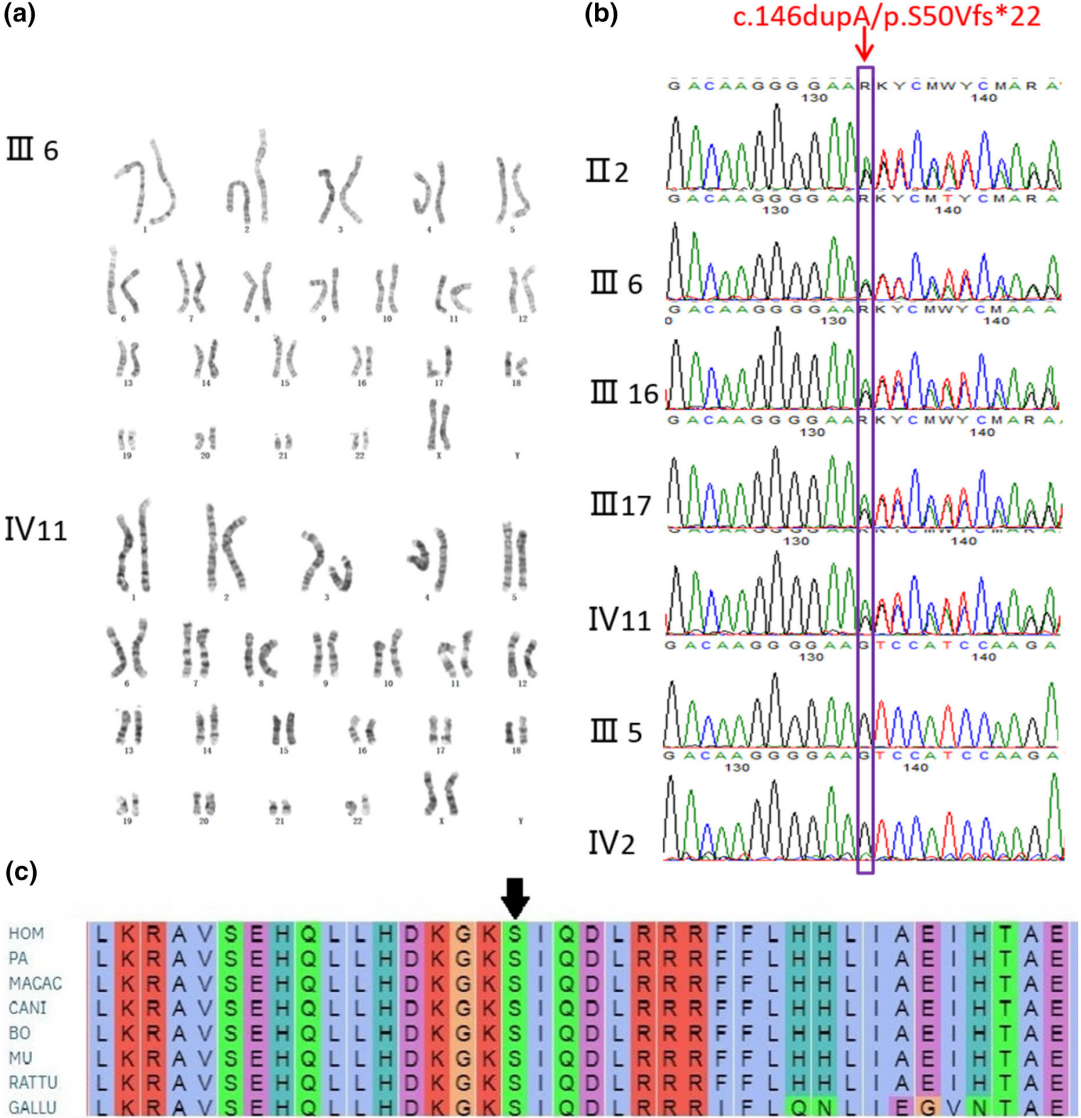
Chromosome karyotype analysis and sequencing of *PTHLH* exons. (a) No obvious chromosome aberration was found in proband (IV11) and patient III6. (b) Direct sequencing of *PTHLH* exons showing heterozygous c.146dupA, p.S50Vfs*22 mutation identified in BDE affected individuals (II2, III6, III16, III17, IV11) but not in normal family members (III5, IV2). (c) Evolutionary conservation of the cluster across multiple species.

Both the proband and patient III6 showed normal chromosomal karyotypes (Figure 3). The exome of the proband revealed a novel heterozygous duplication of an A at position c.146 in exon 4 of *PTHLH* gene (c.146dupA, p.S50Vfs*22) that led to a premature stop codon, and the mutation was validated by Sanger sequence subsequently. All the enrolled BDE affected individuals including proband, II2, III6, III 16, and III17 revealed the c.146dupA, (p.S50Vfs*22) mutation; alternatively, the normal individuals in the pedigree exhibited normal sequencing results (Figure 3).

## 4 DISCUSSION

BDE includes variable shortening of the metacarpals, metatarsals, and/or phalanges (Pereda et al., 2013). The genetic etiology is complex and not fully elucidated. Up to now, two cases of balanced translocation including *t*(8; 12) (q13; p11.2) and *t*(4; 12) (q13.2–13.3; p11.2) have been reported by Maass et al. (2010, 2012); both translocations caused downregulation of the *PTHLH* gene by disrupting the cis‐regulatory and in trans‐regulatory landscape. Compared to the translocation, more microdeletion/duplication syndromes are determined, Klopocki et al. (2010) detected 907 kb genomic microdeletion on chromosome 12p which affected six genes. Huang et al. (2019) identified a 3.06‐Mb deletion at 12p11.22–12.1, that region encompasses 23 annotated genes. Flottmann et al. (2016) revealed a 70‐kb duplication on chromosome 12p11.22. The above chromosome aberrations were encompassing gene *PTHLH*, which inferred *PTHLH* aberration is probably a cause of BDE. In addition to microdeletion/duplication segments relating to *PTHLH*, other aberrations have also been reported, such as 800‐kb Interstitial deletion on 2q37.3q37.3 (Villavicencio‐Lorini et al., 2013) and 3‐Mb microduplication in region 6p25 (Fontana et al., 2017). However, no chromosome translocation or microdeletion/duplication was identified in our research.

Through further whole exome sequencing, we identified a novel mutation (c.146dupA, p.S50Vfs*22) in the *PTHLH* gene, which occurred in a Chinese four‐generation family presenting brachydactyly type E symptoms. The identified variant was located in a highly conserved sequence and co‐segregated with the phenotype in this family.

**FIGURE 4:**
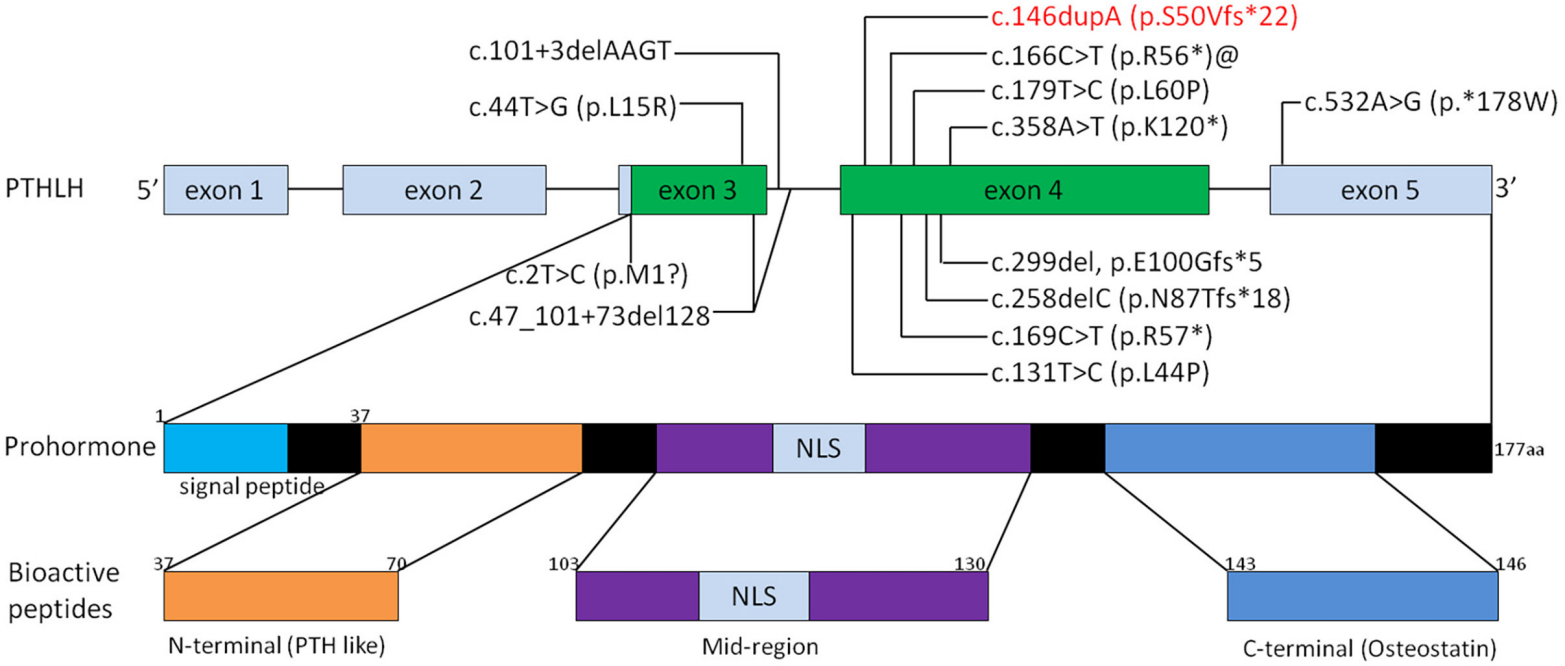
Schematic representation of the *PTHLH* gene and its proteolytic processing pattern into bioactive peptides. Prohormone has 177 amino acids. Bioactive peptides include mature N terminus (amino acids 37–70), mid‐region (containing the nuclear localization signal) (amino acids 103–130), and C‐terminal (amino acids 143–146) peptides. Known mutations reported in literature are denoted in the gene structure and mutation found in our study is reported in red. @ highlights recurrent mutations. NLS, nuclear localization signal.

The *PTHLH* (NM_198965.1) gene comprising of five exons is located at human chromosome 12p11.22 (Yasuda et al., 1989). It encodes for parathyroid hormone‐related protein (PTHrP) which mainly secreted by chondrocytes, perichondrial cells, and osteoblasts, and plays an important role in endochondral bone development (Hiremath & Wysolmerski, 2013; Strewler, 2000). The nascent translation product of PTHrP goes through several proteolytic cleavage processes and generates three bioactive peptides encompassing the NH2‐terminal peptide (Figure 4), which has PTH‐like and growth regulatory activities; the midregion domain, regulating calcium transport and cell proliferation and containing the nuclear localization signal (NLS); and the COOH‐terminal domain, also named osteostatin, modulating osteoclast activity (Kronenberg, 2006). PTHrP activates the same receptor as PTH, during skeletal development, PTHrP binds PTH/PTHrP receptor and activates Gsa‐cAMP‐PKA‐PDE4D signaling pathway, which in turn promotes chondrocytes proliferating and undifferentiation suppressing of *p57* and *Runx2* and phosphorylation of *SOX9*, thereby increasing the pool of proliferating chondrocytes (Guo et al., 2006; Huang et al., 2001). And its downstream targets *ADAMTS‐7* and *ADAMTS‐12* are highly expressed in the fibroblasts and seem to play an important role in chondrogenesis (Bai, Wang, Kong, et al., 2009; Bai, Wang, Luan, et al., 2009). Additionally, the *IHH* is a negative feedback regulator of *PTHLH* and can control the expression level of PTHrP to regulate the hypertrophic differentiation of chondrocytes (van Donkelaar & Huiskes, 2007; Vortkamp et al., 1996). Knockout of *PTHLH* in the mouse results in a lethal dyschondroplasia phenotype including short‐limbed dwarfism (Karaplis et al., 1994). However, the overexpression of *PTHLH* also induces short‐limbed dwarfism and a delay in endochondral ossification (Weir et al., 1996). As a result, the disruption of the above‐mentioned pathways and haploinsufficiency of *PTHLH* will lead to complex dysplasia and growth disorder of bone.

Till now, consistent with our finding, a host of mutations in *PTHLH* gene was also reported as pathogenic causes in literatures studying BDE. Klopocki et al. (2010) identified p.L44P, p.L60P, p.K120*, and p.*178 W firstly in 2010 and proposed that loss‐of‐function mutations in *PTHLH* caused BDE with short stature. Subsequently, p.L15R, p.R56*, p.N87Tfs*18, p.R57*, p.Met1?, and p.E100Gfs*5 mutation in exons were identified (Bae et al., 2018; Elli et al., 2021; Jamsheer et al., 2016; Pereda et al., 2017; Wang et al., 2015). Additionally, two intragenetic novel alterations involving intron (c.101 + 3delAAGT, c.47_101 + 73del128) were also identified (Thomas‐Teinturier et al., 2016). However, in all reported patients, the clinical features were variable interfamilially, even if the mutations co‐segregated with the disease and had complete penetrance. The mild symptom of BDE may induce the shortening of the fourth metacarpals and metatarsals isolatedly; meanwhile, the severe phenotype affected all the digits. What is noteworthy is that the p.R56 mutation occurred recurrently in three reports (Figure 3), although the BDE is rare. With the same mutation, Jamsheer et al. (2016) showed the patient with facial dysmorphism and short second, third, and fifth fingers and short 3–5 toes. While Pereda et al. (2017) illustrated shortening of all the metacarpals and shortening of third, fourth, and fifth metatarsals. The patients illustrated by Elli et al. (2021) manifested shortening of third, fourth, and fifth metacarpals and, additionally, third and fourth metatarsals and second, third, fourth, and fifth phalanges, associated with short stature. In our study, although all the patients carried the same mutation, the phenotype was not consistent which further signified the intrafamilial variability of BDE. The proband presented with shorted metacarpals and phalanges of both hands and shortened fourth and fifth toes in both feet. However, patient III6 exhibited shortening of third, fourth, and fifth metatarsals in right foot. Meanwhile, the patient II2 only had short fourth metacarpals in both hands. Our gathered data also showed that all the c.146dupA mutation carried individuals had short stature (−2 SD), including II2, III6, III16, III17, IV11, but the unaffected members in the pedigree had normal height, which was consistent with the previous literatures manifested that most affected individuals with *PTHLH* mutations had short stature (Elli et al., 2021; Klopocki et al., 2010; Thomas‐Teinturier et al., 2016). The short height of the affected individuals was probably caused by the premature closure of growth plate (Karaplis et al., 1994). In contrast to previous studies, the patients described in this research did not manifest delayed tooth eruption and/or oligodontia, facial abnormalities, or abnormal mammary glands (Jamsheer et al., 2016; Klopocki et al., 2010; Thomas‐Teinturier et al., 2016).

Furthermore, the mutation (p.S50Vfs*22) was expected to lead to premature termination of protein, which resulted in a truncation of the protein within N terminus. Because the mutation was located more than 50 bp away from the 3′ of the penultimate exon, thereby most probably subjected to nonsense‐mediated decay (Popp & Maquat, 2013). To determine the precise reduction in *PTHLH* mRNA, we conducted QRT‐PCR, however, no legitimate expression of the transcripts in blood was detected, which was consistent with previous reports (Jamsheer et al., 2016; Thomas‐Teinturier et al., 2016). In the future, the exact mechanism leading to BDE and short stature caused by this novel mutation should be investigated intensively and comprehensively.

In conclusion, we reported a novel mutation c.146dupA, p.S50Vfs*22 of *PTHLH* gene identified in a four‐generation family with BDE and short stature. The mutation led to a premature termination of protein and was proved to be pathogenic, which broadened the mutation spectrum of *PTHLH* in BDE.

## AUTHOR CONTRIBUTIONS

Cheng Hongbo and Zhang Xiangxin were responsible for the study design. Sun Jian and Yang Nian were responsible for sequencing analysis and drafting of manuscript. Xu Zhengquan was responsible for clinical diagnosis and clinical data recruitment.

## CONFLICT OF INTEREST STATEMENT

None.

## Data Availability

The data that support the findings of this study are available from the corresponding author upon reasonable request.
